# Endothelial Function in Patients with Hematologic Malignancies Undergoing High-Dose Chemotherapy Followed by Hematopoietic Stem Cell Transplantation

**DOI:** 10.1007/s12012-015-9324-0

**Published:** 2015-04-09

**Authors:** Małgorzata Poręba, Paweł Gać, Lidia Usnarska-Zubkiewicz, Witold Pilecki, Kazimierz Kuliczkowski, Grzegorz Mazur, Małgorzata Sobieszczańska, Rafał Poręba

**Affiliations:** Department of Pathophysiology, Wroclaw Medical University, Marcinkowskiego 1 Street, 50-368 Wrocław, Poland; Department of Hematology, Blood Neoplasms and Bone Marrow Transplantation, Wroclaw Medical University, Pasteur 4, 50-367 Wrocław, Poland; Department of Internal Medicine, Occupational Diseases and Hypertension, Wroclaw Medical University, Borowska 213, 50-556 Wrocław, Poland

**Keywords:** Ultrasonography, Brachial artery, Endothelium, Hematopoietic stem cell transplantation

## Abstract

The aim of the study was to examine endothelial function in patients with hematological malignancies treated with high-dose chemotherapy followed by hematopoietic stem cell transplantation. The studies were conducted on 43 consecutive patients qualified for HSCT following high-dose chemotherapy based on the current standards. Then, due to exclusion criteria, a group of 38 patients were chosen for further investigations. Evaluation of endothelial function by means of flow-mediated dilatation (FMD) was conducted in patients with hematological malignancies before HSCT (test A) and after HSCT (test B). Brachial artery diameter (BAD) after occlusion, change in BAD and FMD were significantly lower after HSCT as compared to the results obtained before the transplantation (*p* < 0.05). The regression analysis indicated that administration of fludarabine and cytarabine, and also higher blood concentrations of creatinine represented risk factors for the impairment of endothelial function expressed as decreased FMD value. In patients with hematopoietic malignancies treated with HSCT, endothelial function assessed by the flow-mediated dilatation was impaired after chemotherapy and stem cell administration.

## Introduction

Hematopoietic stem cell transplantation (HSCT) is an important therapeutic strategy in many hematologic malignancies and significantly prolongs survival in patients. In hematological malignancies, sustained remissions and even cures may be achieved in certain patients by use of HSCT following the high-dose chemotherapy (HDC) [1, 2].

Nevertheless, clinical observations suggest that cardiovascular complications may appear in some patients in the course of peripheral blood stem cell transplantation and also there is a serious risk of developing some long-term complications. Until now, the detailed range of possible cardiovascular complications and potential cardiotoxic effects of the stem cell administration in individual groups of patients remains unknown. Our previous studies showed that in patients with hematopoietic malignancies undergoing HSCT, after chemotherapy and stem cell administration, the decreased heart rate variability and heart rate turbulence were observed [3].

It is known that impaired endothelial function plays an important role in the pathophysiology of atherosclerotic cardiovascular diseases [4]. Although, endothelial dysfunction is an early-stage change within the organism, it probably leads to further complications and results in specific diseases. Endothelial function can be evaluated by means of biochemical or functional methods. Assessment of flow-mediated dilatation (FMD) is one of the possible functional and noninvasive methods. FMD is the assessment of brachial artery reactivity to hyperemia induced by compression [5].

This study aimed at evaluation of endothelial function by means of FMD in the patients with hematological malignancies treated with high-dose chemotherapy in the course of hematopoietic stem cell transplantation.

## Materials and Methods

All procedures followed were in accordance with the ethical standards of the responsible committee on human experimentation and with the Declaration of Helsinki. Informed consent was obtained from all patients included in the study.

### Study Population

We included 43 consecutive patients in the study group fulfilling the following criteria: age ≥18 years, the diagnosed blood cancer, complete remission status, and qualification for the HSCT procedure following high-dose chemotherapy. Then, five patients were excluded from the study due to comorbidities that met the exclusion criteria: diabetes mellitus, ischemic heart disease, previous cerebral stroke and arterial hypertension. Eventually, a group of 38 patients was obtained as a final study group. General characteristics of the studied group are presented in Table 1.

**Table 1:** Clinical characteristics of the study group

Age (years)	42.88 ± 13.49
*Gender (%/n)*	
Men	60.53/23
Women	39.47/15
Body mass index (BMI ) (kg/m^2^)	24.57 ± 3.18
Overweight (%/n)	15.79/6
Obesity (%/n)	0.00/0
*Cigarette smoking (%/n)*	
Currently	0.00/0
In the past	23.68/9
*Blood neoplasms (%/n)*	
Acute myeloid leukemia	28.95/11
Hodgkin’s lymphoma	18.42/7
Multiple myeloma	18.42/7
Acute lymphoblastic leukemia	15.79/6
Non-Hodgkin’s lymphoma	13.16/5
Chronic myeloid leukemia	5.26/2

### Study Protocol

At first, patients undergoing the autologous HSCT (22 patients) had the procedure for mobilization and collecting stem cells. Afterward, they were given high-dose chemotherapy, and after the time period defined in treatment protocol patients, stem cells were administered. In the remaining group of 16 patients, allogeneic HSCT was performed. All patients before HSCT procedure were in a clinically good condition, 0–1 expressed in Eastern Cooperative Oncology Group (ECOG) performance status categories, without clinical signs of infection, and with normal laboratory tests. Results of basic laboratory tests are presented in Table 2.

**Table 2:** Laboratory characteristics of patients before HSCT

RBC (mln/ml)	3.85 ± 0.49
Hematocrit (%)	36.04 ± 4.43
Hemoglobin (g/dl)	11.81 ± 2.34
WBC (×10^2^/L)	6.13 ± 3.22
Platelet count (×10^2^/L)	159.25 ± 85.15
Sodium (mmol/l)	14.2.06 ± 2.66
Potassium (mmol/l)	4.08 ± 0.38
Prothrombin activity (%)	94.90 ± 10.52
Fibrinogen (mg/dl)	380.71 ± 72.27
APTT (s)	33.80 ± 5.75
Urea (mg/dl)	31.58 ± 11.01
Creatinine (mg/dl)	0.87 ± 0.24
Uric acid (mg/dl)	5.29 ± 1.36
Glucose (mg/dl)	96.00 ± 24.26
Serum protein (g/dl)	6,80 ± 0.63
Albumin (g/dl)	4.10 ± 0.42
Total cholesterol (mg/dl)	194.09 ± 36.80
Triglycerides (mg/dl)	128.67 ± 46.50
Aspartate aminotransferase (U/l)	19.81 ± 7.95
Alanine aminotransferase (U/l)	29,21 ± 24.77
Lactate dehydrogenase (U/l)	178.83 ± 29.39
Total bilirubin (mg/dl)	0.79 ± 0.69
C-reactive protein (mg/l)	4.69 ± 3.18

In all participants of the study, the examination of vascular endothelium with the use of FMD was performed twice. The first examination was prior to the HSCT procedure (test A). Then, the patients were administered a HDC, the type of which was appropriate to the diagnosed malignancy, according to patient’s body weight or body surface area. The type of cytostatic drugs and the frequency of their applying in high-dose chemotherapy as well as the frequency of total body irradiation in the study group are shown in Table 3. The second test evaluating the endothelial function was performed after the HSCT, around 20 days following administration of chemotherapy (mean 22.46 ± 3.19 days) (test B). The time of the second examination was dependent on the clinical condition of the patient, that is, when patient was at good condition, without severe life-threatening leucopenia.

**Table 3:** Cytostatics used in high-dose chemotherapy (HDC) and total body irradiation (TBI) before HSCT

	Percentage/number of patients
*Cytostatic (%/n)*	
Melphalan	57.89/22
Carmustine—BCNU	34.21/13
Etoposide	34.21/13
Cytarabine—Ara-C	31.58/12
Cyclophosphamide	28.95/11
Busulfan	26.32/10
Fludarabine	13.16/5
Total body irradiation (%/n)	21.05/8

The precise description of the applied protocol of the study was presented in previous article of the authors, which was concentrating on the heart rate variability and heart rate turbulence in patients with hematologic malignancies undergoing high-dose chemotherapy followed by hematopoietic stem cell transplantation [3].

### FMD Measurements

**Fig. 1:**
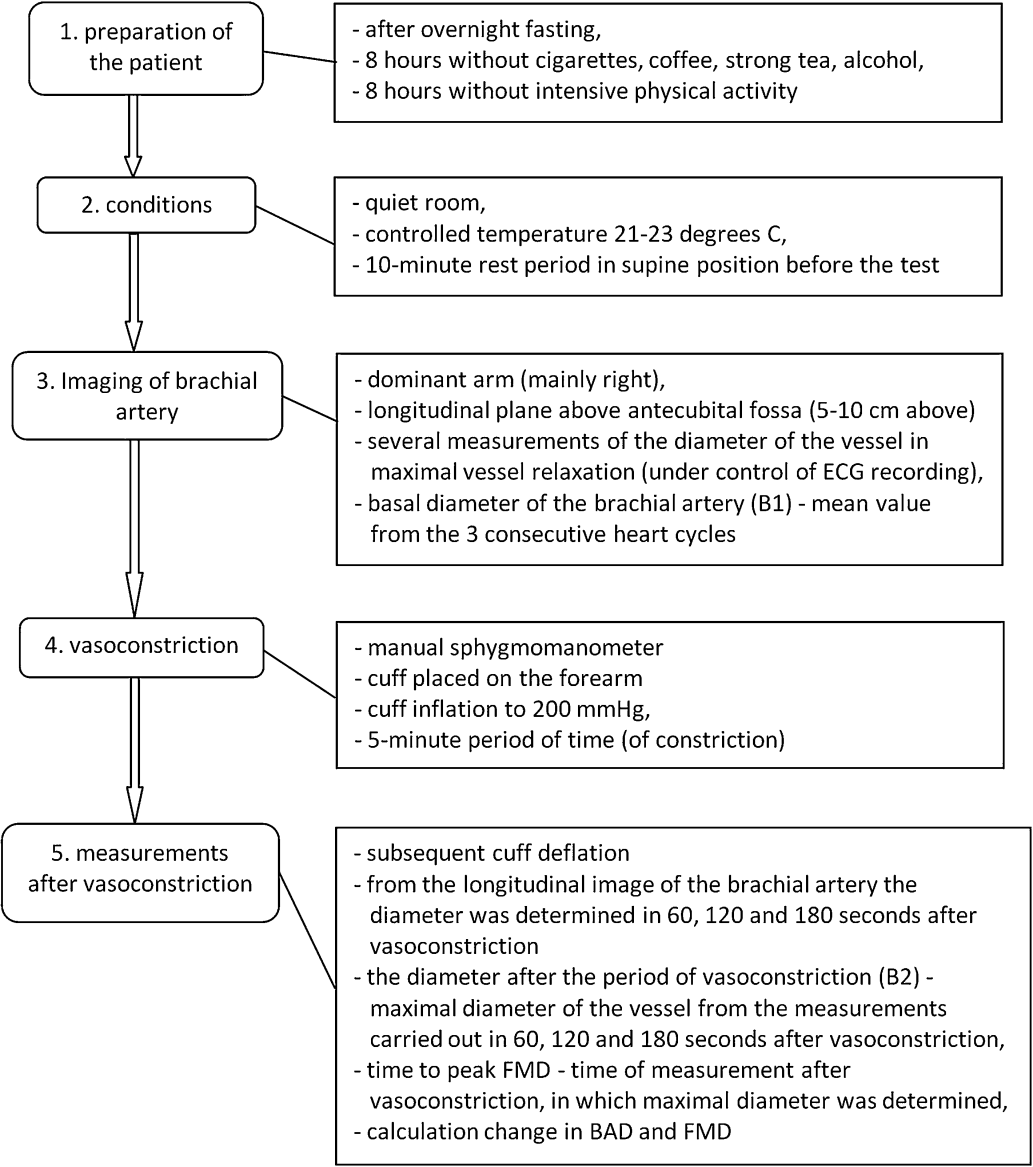
Details on the methods of FMD measurements

FMD measurements were taken using an Aloka SSD-5500 ultrasound device equipped with a 7.5-MHz linear transducer according to guidelines for the ultrasound assessment of endothelial-dependent flow-mediated vasodilation of the brachial artery [5]. The basal diameter of the brachial artery was determined (B1), as well as, the diameter after the period of vasoconstriction (B2), the difference of the brachial artery diameter before and after vasoconstriction (change in BAD: B2-B1) and the flow-mediated vasodilatation (FMD) of the brachial artery with the use of the formula: FMD (%) = (B2 − B1/B1) × 100. Details on the methods of FMD measurements are presented in the Fig. 1. The estimated reproducibility of FMD is about 90 %. The repeatability of FMD was estimated comparing the difference between two measurements taken in each patient in 15 min periods and taking the limits of compatibility of arithmetic mean ± 2 × standard deviations into account. About 90 % of the obtained differences of the results of FMD were within the limit of compatibility.

### Statistical Analysis

Statistical analysis was conducted using STATISTICA 9 software (StatSoft, Polska). For quantitative variables, arithmetic means (X) and standard deviations (SD) of estimated parameters were calculated in the analyzed groups. Distribution of variables was examined using tests of Lilliefors and W-Shapiro–Wilk. For dependent qualitative variables of the normal distribution, the *t* test for linked variables was applied. In cases of quantitative dependent variables with the distribution distinct from normal, the pair sequence test of Wilcoxon was applied. Results for qualitative variables were expressed in percentages. For qualitative dependent variables, statistical analysis involved McNemar’s test or Cochran’s *Q* test. In order to define a relationship between the studied variables, analysis of multivariable regression was performed. Parameters of the model obtained in regression analysis were estimated using the technique of least squares. Results at the level of *p* < 0.05 were assumed to be of statistical significance.

## Results

Comparing measurements of FMD taken after HSCT procedure (test B) and before it (test A), there was no statistically significant difference in B1 (basal diameter of the brachial artery) value. However, significant differences were found in other FMD parameters, Table 4. The value of B2, and change in BAD and FMD were significantly lower in test B, as compared to the results of test A (*p* < 0.05).

**Table 4:** Brachial artery diameter and flow-mediated dilatation in the study group

	Test A	Test B	*p*
Baseline BAD: B1 (mm)	3.83 ± 0.57	3.84 ± 0.58	ns
BAD after occlusion: B2 (mm)	4.22 ± 0.62	4.09 ± 0.63	*p* < 0.01
Change in BAD: B2-B1(mm)	0.39 ± 0.10	0.25 ± 0.09	*p* < 0.001
FMD (%)	10.14 ± 2.27	6.43 ± 2.03	*p* < 0.001
Time to peak FMD (s)	85.26 ± 35.92	93.16 ± 36.10	ns

The multivariable stepwise backward regression analysis, performed in the whole study group, provided the following model: ΔFMD = 3.32 + 4.66 fludarabine + 2.27 cytarabine + 1.02 creatinine ± 0.69, where ΔFMD was defined as difference between FMD in test A and test B. Simultaneously, it was taking into account the basic clinical parameters (age, gender, BMI, blood cell count and biochemical parameters), the type of hematopoietic malignancy (acute myeloblastic leukemia, Hodgkin’s lymphoma, multiple myeloma, acute lymphoblastic leukemia, nonHodgkin’s lymphoma and chronic myeloblastic leukemia), the type of administered cytostatic drugs for high-dose chemotherapy in the course of HSCT procedure (i.e., melphalan, carmustine, etoposide, cytarabine, cyclophosphamide and busulfan), total body irradiation, the type of stem cell transplantation (auto, allo) and time between test A and test B.

The obtained models indicated that in the group of patients with hematopoietic malignancies who were given high-dose chemotherapy in the course of hematopoietic stem cell transplantation, the administration of fludarabine and cytarabine, and also the initial higher blood concentrations of creatinine represented independent risk factors for the impairment of endothelial function (expressed by elevated difference between FMD in the test A and FMD in the test B). Results of estimations of the models obtained in multivariable stepwise backward regression analysis are presented in Table 5.

**Table 5:** Results of estimation for the final models obtained in the multivariate backward stepwise regression analysis

Model for ΔFMD (%)			
	Fludarabine	Cytarabine—Ara-C	Creatinine (mg/dl)
Regression coefficient	4.661	2.268	1.021
SEM of Rc	0.471	0.267	0.548
*P* value	0.001	0.001	0.043
*P* value for the model	*p* < 0.002		

ΔFMD—difference between FMD in test A and FMD in test B intercept: 3.317

Fludarabine and Cytarabine—Ara-C—nominal variables, where 1: yes, 0: no

SEM of Rc—standard error of the mean of regression coefficient

Partial models were defined to determine which parameters used in the obtained model are of the highest significance within the aspect of impairment of endothelial function in patients with hematopoietic malignancies who experienced high-dose chemotherapy and hematopoietic stem cell transplantation. Parameters of partial models are shown in Table 6.

**Table 6:** Parameters of partial models in multivariate backward stepwise regression analysis

Partial models for ΔFMD (%)							
Parameter	Regression coefficient	SEM of RC	Intercept	SEM of intercept	SEM of estimate	*R* ^2^	*p* value
Fludarabine	4.422	0.725	3.239	0.235	1.372	0.494	0.001
Cytarabine—Ara-C	1.401	0.641	3.262	0.360	1.838	0.293	0.015
Creatinine (mg/dl)	1.393	1.155	2.502	1.044	1.593	0.145	0.024

Fludarabine and cytarabine—Ara-C—nominal variables, where 1: yes, 0: no

SEM of Rc—standard error of the mean of regression coefficient

Basing on the partial regression analysis, it has been shown that the administration of fludarabine has the highest significance (the highest value of R2 in partial models = 0.494; with the lowest *p* value = 0.001), in the aspect of impairment of endothelial function in this group of patients.

## Discussion

Endothelium regulates the vascular tone and influences hemostasis and vascular permeability, taking responsibility for the proper delivery of blood to tissues [6]. Among noninvasive methods assessing endothelial function that are currently used, flow-mediated vasodilatation of the brachial artery induced by reactive hyperemia is a relatively well-known and valuable technique.

According to Celermayer et al. [7], disturbances in vascular biology provide a basis to identify individuals at potential risk of cardiovascular events. Some authors claim that evaluating of endothelial function with the use of FMD may offer a noninvasive measure of preclinical cardiovascular risk in populations with and without obvious risk factors [8, 9]. Preclinical detection of endothelial dysfunction may enable to plan interventions and monitoring at an early stage of the disease. FMD, a useful method of determination of endothelial function, was found to be an independent predictor for further cardiac events in patients after myocardial infarction [10, 11].

Chemotherapy is a widely used therapeutic approach for many malignancies; however, its efficacy is limited by toxicity, including cardiotoxicity leading to several complications, among which cardiomyopathy and heart failure are the most severe forms [12].

A number of studies have been done demonstrating effects of chemotherapy on endothelial function in cancer survivors. It was observed that adult survivors of childhood acute lymphoblastic leukemia were at risk of impaired FMD [13]. Moreover, survivors of leukemia had lower carotid distensibility and compliance, indicating increased arterial stiffness, when compared to controls in the study of Dengel et al. [14], indicating increased risk of premature atherosclerosis and cardiovascular disease. Therefore, authors suggested monitoring of cardiovascular risk factors in such groups of patients. The study showed in a large sample of children that leukemia survivors presented significantly decreased measures of vascular function in both the brachial and carotid arteries [15]. Authors also mentioned studies of Herceg-Cavrak et al. exploring the method of the pulse wave velocity, a marker of arterial stiffness, in children and adolescents after anthracycline treatment. In spite of some limitations, it was observed that pulse wave velocity was significantly increased in cancer survivors [16]. Additionally, in a study assessing toxicity from anthracyclines in pediatric cancer patients, brachial artery reactivity was calculated and it was proved that anthracyclines caused endothelial dysfunction [17]. Similar results were shown by other authors in a group of long-term survivors of acute lymphoblastic leukemia in whom impaired FMD response was found [18].

Cardiotoxicity caused by chemotherapeutic regimens including peri-transplantation period is currently an important issue. Unfortunately, echocardiography fails to detect more subtle alterations in the heart [12]. The novel strategy such as flow-mediated vasodilatation is an innovative idea, and some changes in endothelial function could be a sign of an early-stage changes in cardiovascular system.

We have made an attempt to evaluate the potential endothelial dysfunction as an early-stage damage in cardiovascular system after HSCT with the use of high-dose chemotherapy. Up till now, there have been not many reports where FMD was measured as a method of monitoring cardiotoxicity. Nagy et al. [19] observed the decreased values of FMD in short time after bolus of doxorubicin in patients with lymphomas, explaining the phenomenon by the abundant oxidative stress.

Transplantation is based not only on chemotherapy, but also includes the following administration of stem cells. The probable adverse effects of this procedure are mainly caused by cardiotoxicity of drugs; however, the influence of stem cells is yet not well investigated.

Our study showed the decrease in FMD after HSCT procedure about 22 days after transplantation. Contrary to the studies of Nagy et al. [19], our tests were carried out not hours after chemotherapy, but a couple of days, when patients were in good condition after transplantation. Additionally, multivariable stepwise backward regression analysis discovered that some cytotoxic drugs such as fludarabine and cytarabine represented the independent risk factors for the impairment of endothelial function (expressed by elevated difference between FMD in the test A and FMD in the test B). Moreover, the initial higher blood concentrations of creatinine also was indicated as risk factor for developing endothelial dysfunction measured by FMD. In other words, basing on our study, it could be suggested that the dysfunction of endothelium is more frequently present in patients after HSCT with impairment of renal function. Moreover, some other reports support the idea that FMD was decreased in patients with renal function impairment [20, 21].

For the patients suffering from blood neoplasms, finding the appropriate treatment giving the chance for cure or at least potentially good quality prolonged life is a basic matter. Generally, the procedures of stem cell or bone marrow transplantations give patients with various blood neoplasm hope for achieving these goals. Our study attempts to check the potential and possible short-term complications caused by transplantations on the level of minor changes, that is, within endothelium. Although methods of treatment of blood cancers are known, they are not perfect, and there are some limitations within the used regimens, including short- and long-term complications. Anticancer therapy is lifesaving, so in this perspective some side effects may be accepted. However, we should remember that diseases of the cardiovascular system are globally the most common death causes, and a lot of patients may be more prone to develop them in future or they may theoretically be accelerated or induced by the treatment. The endothelial dysfunction is the first change in the organism that may predict cardiovascular problems. In this context, the improvements of the used treatment should be planned not only with the idea of the highest efficacy, but simultaneously, in order to avoid the unnecessary negative effect on the endothelium, which is the key organ involved in pathogenesis of cardiovascular diseases.

Regarding limitations of the study, the simplicity of the study protocol, basing on only two measurements of the studied parameters before and after high-dose chemotherapy and stem cell transplantation, is one of them. However, this limitation resulted directly from the restrictions that must have been obeyed at the transplantation center, especially after chemotherapy, when patients were at the highest risk of infection. The studies were planned to be as convenient for the patients, as possible.

As to the other limitations, we have designed the study to compare the specific parameters in patients with blood neoplasms before and after transplantation procedure, and, from the traditional point of view, there is no control group. Due to the specificity of the study group, it could be moderately difficult to find a suitable group of healthy people with similar anthropometric, clinical and laboratory parameters, meeting similar exclusion criteria.

Limitations of the study include the difficulty in separating cardiotoxicity of drugs and the influence of stem cells. An additional test carried out after high-dose chemotherapy and before transplantation could help in this case. However, as mentioned above, in the transplantation center, patients were temporary isolated.

The next limitation is not taking into account previous chemotherapy; however, in our opinion due to the diversity of the group and different types of chemotherapy used, it would make the article too large. The chemotherapy was different for each disease, based on standards, and anthracyclines were administered not exceeding the accepted maximum cumulative doses expressed in mg/m^2^. Simultaneously, we agree that it is possible that there may be a potential influence of the previous chemotherapy on the baseline FMD in our study.

Eventually, although our study includes some limitations, simultaneously it presents some unique and novel information. As the endothelial dysfunction is thought to be an early change, it would be reasonable to perform a longer follow-up research in such a population of patients suffering from hematologic neoplasms treated by stem cell transplantation.

## Conclusions

1. In patients with hematopoietic malignancies undergoing high-dose chemotherapy followed by hematopoietic stem cell transplantation, endothelial function assessed by the flow-mediated dilatation was impaired in the tests performed after completion of the procedure, as compared to the tests performed directly before high-dose chemotherapy.
2. In the group of patients with hematopoietic malignancies, in the course of hematopoietic stem cell transplantation, application of fludarabine and cytarabine—Ara-C—and higher initial creatinine blood concentrations represent independent risk factors for the impairment of endothelial function expressed as decreased FMD value.
3. Although, at the first sight, endothelial dysfunction found in patients with hematologic cancers after transplantation in a short-term observation period did not change the treatment regimens, as it seems to be a minor concern in seriously ill individuals, further studies are needed to check whether in long-term observation patients in which the endothelial dysfunction was present may be at higher risk of the developing cardiovascular events and deaths caused by them.
